# Comprehensive Analysis of the Naturally Processed Peptide Repertoire: Differences between HLA-A and B in the Immunopeptidome

**DOI:** 10.1371/journal.pone.0136417

**Published:** 2015-09-16

**Authors:** Ingrid M. M. Schellens, Ilka Hoof, Hugo D. Meiring, Sanne N. M. Spijkers, Martien C. M. Poelen, Jacqueline A. M. van Gaans-van den Brink, Kees van der Poel, Ana I. Costa, Cecile A. C. M. van Els, Debbie van Baarle, Can Kesmir

**Affiliations:** 1 Laboratory of Translational Immunology, Department of Immunology, University Medical Center Utrecht, Utrecht, the Netherlands; 2 Department of Internal Medicine and Infectious Diseases, University Medical Center Utrecht, Utrecht, the Netherlands; 3 Theoretical Biology and Bioinformatics, Utrecht University, Utrecht, The Netherlands; 4 Centre for Immunology of Infectious Diseases and Vaccines, National Institute for Public Health and the Environment, Bilthoven, The Netherlands; 5 Institute for Translational Vaccinology (Intravacc), Bilthoven, The Netherlands; Johns Hopkins University, UNITED STATES

## Abstract

The cytotoxic T cell (CTL) response is determined by the peptide repertoire presented by the HLA class I molecules of an individual. We performed an in-depth analysis of the peptide repertoire presented by a broad panel of common HLA class I molecules on four B lymphoblastoid cell-lines (BLCL). Peptide elution and mass spectrometry analysis were utilised to investigate the number and abundance of self-peptides. Altogether, 7897 unique self-peptides, derived of 4344 proteins, were eluted. After viral infection, the number of unique self-peptides eluted significantly decreased compared to uninfected cells, paralleled by a decrease in the number of source proteins. In the overall dataset, the total number of unique self-peptides eluted from HLA-B molecules was larger than from HLA-A molecules, and they were derived from a larger number of source proteins. These results in B cells suggest that HLA-B molecules possibly present a more diverse repertoire compared to their HLA-A counterparts, which may contribute to their immunodominance. This study provides a unique data set giving new insights into the complex system of antigen presentation for a broad panel of HLA molecules, many of which were never studied this extensively before.

## Introduction

The function of Human leukocyte antigen (HLA) class I molecules is to present intracellular peptides to CD8+ T cells. Cytosolic and nuclear proteins as well as proteins of intracellular pathogens are degraded by (cytosolic) proteases, and the resulting peptide fragments are transported into the endoplasmic reticulum (ER) by the transporter associated with antigen processing (TAP) complex. In the ER the peptide fragments bind to available HLA class I molecules after which the peptide-HLA (pHLA) complexes leave the ER and reach the cell surface [1]. Each nucleated cell can express several hundred thousand copies of up to six different classical HLA class I molecules, each molecule containing a single peptide [2]. The repertoire of peptides presented by HLA molecules on the cell surface is often referred to as the immunopeptidome [3,4]. The HLA gene cluster is the most polymorphic cluster in the human genome, with already over 6500 different geneproducts described for the three major groups of HLA class I molecules (HLA-A, HLA-B, and HLA-C) [5], all having unique peptide binding properties. Therefore, each individual, depending on the inherited combination of up to six different HLA class I molecules, may present a unique immunopeptidome and therefore respond differently to infectious diseases, inflammatory conditions, autoimmune diseases and malignancies.

Many genetic associations between HLA class I molecules and infectious diseases have been reported, including but not limited to, HIV/AIDS, hepatitis, leprosy, tuberculosis, malaria, leishmaniasis and schistosomiasis (reviewed in [6]). HLA-B molecules, the most polymorphic of the HLA allotypes, are frequently associated with disease outcome, either beneficial or detrimental. Moreover, HLA-B restricted T-cell responses have been shown to be immunodominant as compared to HLA-A and -C restricted responses, both within individuals [7–9], and at a population level [10]. This immunodominance is not well understood but might be explained by potential differences in the presentation of peptides between HLA loci or by differences in the quality and/or quantity of the restricted CD8+ T-cell responses.

As the association between HLA-B and disease outcome surpasses single infectious agents, we hypothesised that general characteristics of HLA-B molecules, e.g. the diversity and/or the abundance of the peptides presented at the cell surface, may be driving these associations. Therefore, we here analysed the repertoire of naturally processed peptides presented on 4 different B lymphoblastoid cell-lines (BLCL), together expressing 5 different HLA-A, 6 HLA-B and 5 HLA-C molecules. The immunopeptidomes of these molecules have been analysed before and after infection with measles virus (MV) to investigate potential locus-specific preferences as well as changes upon infection in the presented repertoire. MV was chosen as a model pathogen, for several reasons. First, MV infects B cells, also in large scale cultures, being of use in immunopeptidome studies [11]. Second, T-cell mediated immunity has been shown to be important for clearance of MV infection and protection against re-infection. And third, several HLA-B molecules have been shown to be associated with higher immune levels after MV vaccination [12,13].

Peptide-HLA complexes were affinity-purified from BLCL, after which peptides were separated from their HLA molecules using acid elution. NanoHPLC-tandem mass-spectrometry of peptide fractions and strict interrogation of the mass spectra against human and viral databases was utilised to *bona fide* identify the naturally processed and presented peptides. Using state-of-the-art HLA-peptide affinity prediction programs, we assigned the identified peptides to HLA class I alleles expressed by the cells and analysed the number and abundance of HLA-locus associated self and viral peptides in order to find features that might underlie HLA-B immunodominance.

## Materials and Methods

### Cell lines and MV infection

BLCL were kindly provided by Dr. T. Mutis (University Medical Center Utrecht, The Netherlands). The HLA-A and B alleles expressed by the four BLCL (given in Table 1) together are present in >70% and >50% of the Caucasian population, respectively, and thus cover a significant part of HLA class I molecules. Each BLCL was grown to 4 x 10^8^ cells in RPMI-1640 medium supplemented with penicillin, streptomycin and 10% heat-inactivated fetal calf serum (FCS) and split over two equal batches. Plaque-purified MV (Edmonston B strain), cultured in Vero cells, was used to infect 2x10^8^ cells of each BLCL at an m.o.i of 0.5 for 48 hr in RPMI-1640 medium supplemented with antibiotics and 2% FCS. Pre- and post-infection cell viability was high. Uninfected and infected cell batches were harvested, washed three times in cold PBS, pelleted, snap-frozen and stored at − 70°C until further use. The study was approved by the Medical Ethical Committee of the University Medical Center Utrecht. No consent was given as the data were analyzed anonymously. All procedures followed were in accordance with the ethical standards of the responsible committee on human experimentation (institutional and national) and with the Helsinki Declaration of 1975, as revised in 2000.

**Table 1:** Number of unique peptides, source proteins and genes eluted per cell line (before and after MV infection combined).

*Cell line*	*HLA-A*	*HLA-B*	*HLA-C*	*Unique peptides*	*Unique proteins*	*Unique genes*
BLCL 1053	A*02:01 A*03:01	B*07:02 B*07:02	C*07:02 C*07:02	1127	919	826
BLCL 1077	A*01:01 A*24:02	B*08:01 B*40:01	C*03:04 C*07:01	1620	1271	1161
BLCL 1090	A*02:01 A*11:01	B*35:01 B*44:02	C*04:01 C*05:01	3120	1932	1701
BLCL 1112	A*02:01 A*02:01	B*15:01 B*44:02	C*03:04 C*05:01	2993	2145	1904
**Total unique**	**5**	**6**	**5**	**7897**	**4344**	**3639**

### Flowcytometry

Small aliquots (2x10^6^) from uninfected and 48 hours MV infected BLCL cultures were harvested and tested on surface expression of MV hemagglutinin and HLA class I molecules. BLCL were washed and stained in parallel using fluorochrome conjugated HLA specific antibodies W6/32 (anti total HLA class I, eBioscience), BB7.2 (anti-HLA-A2, eBioscience), and B1.23.2 (HLA-B,C, eBioscience), or with unconjugated in-house mouse anti MV-hemagglutinin monoclonal antibody 28-10-8. The latter incubation was followed by incubation with GAM-FITC (BD) as a second step. All stainings were performed for 30' at 4°C, and all washing steps were in washing buffer (PBS 1% FCS). Cells were collected and data were acquired using a Fortessa (BD). Analysis was done using FlowJo X software (Tree Star, Inc.). MV infection had a high efficiency (>90%) based on MV-Hemmagglutinin staining and did not affect the overall expression of HLA class I molecules at the cell surface (S1 Fig).

### Isolation of MHC-bound peptides

HLA class I molecules were immunoprecipitated from uninfected and 48 hr MV-infected BLCL as described previously [11] using the HLA class I specific monoclonal antibody W6/32. Briefly, cells were solubilized in buffer containing CHAPS and protease inhibitors. After centrifugation at 10,000×g for 1 h at 4°C supernatants were incubated in succession with three CNBr-activated and Tris-blocked bead formulations, the first non-Ig coupled, the second coupled with normal mouse Ig, and the third coupled with W6/32. pHLA complexes were eluted with 10% acetic acid and peptides were collected by passage over a 10-kDa mw cutoff membrane. Filtrates were concentrated using vacuum centrifugation.

### Nanoscale Liquid Chromatography-Mass spectrometry (nanoscale LC-MS) and epitope identification/semiquantification

Each MHC peptide eluate was reconstituted in 0.1% (v/v) TFA and fractionated into 26 fractions using Strong Cation eXchange (SCX) chromatography on a 200-μm I.D. PolySULFOETHYL Aspartide column (packed in-house), running a linear KCl-gradient starting with water in 0.5% (v/v) acetic acid to 500 mM and 35% (v/v) acetonitrile in 0.5% (v/v) acetic acid. Each fraction was dried by centrifugation under reduced pressure and reconstituted in water containing 5% formic acid and 5% dimethylsulfoxide. Peptide analyses of the fractions (equivalent to ~50·10^6^ cells) were performed on a *nano*scale LC-MS system, essentially as described by Meiring *et al*.[14], comprising a 50-μm and 25-μm I.D. Reprosil-Pur C18-AQ trapping and analytical column, respectively (packed in-house). High resolution MS1 data were acquired on an LTQ-Orbitrap XL mass spectrometer (Thermo Scientific, San Jose, USA) at a resolution of 60,000 FWHM and CID MS/MS fragmentation spectra were acquired on-the-fly in the LTQ mass analyzer on the doubly and triply charged ions only. Peptide identification (with a False Discovery Rate of 5%) was performed with BioWorks 3.3.1 SP1 (Thermo Scientific, San Jose, USA) against the human-annotated and MV-annotated proteins extracted from the UniProtKB/Swiss-Prot database (*Swiss-Prot database version 57*.*10 with taxonomy identifier 'Homo Sapiens'*, www.uniprot.org). No enzyme cleavage specificity was used as a filter during the database search. Moreover, deamidation of N, oxidation of M and phosphorylation of S,T and/or Y were considered as dynamic modifications during the peptide identification process. For semiquantification, Angiotensin-III and Oxytocin (Sigma-Aldrich, St Louis, USA) added to each of the SCX fractions, were used as internal standard peptides for the absolute quantification of expression levels, based on the assumption of equal response factors (counts/mole) for the epitopes and these standard peptides in the MS analysis. These experiments were performed as unique experiments, due to their laborious nature.

### Peptide assignment to HLA class I alleles

We used the W6/32 antibody to isolate HLA-peptide complexes, which has affinity for all three major groups of HLA class I molecules (HLA-A, B and C). For each of the eluted peptides, we applied NetMHC-3.2 [15,16] for the respective BLCL’s HLA-A and -B alleles, and NetMHCpan-2.4 [17,18] for HLA-C alleles. When selecting the BLCL for this study, we took into account that each of the HLA molecules expressed on a specific cell line has a binding motif with no or only little overlap with the binding motifs of the other HLA molecules expressed on the cell line, thereby minimizing the level of cross-presentation (ie the presentation of the same peptide by two or more distinct HLA alleles). Since prediction scores for different HLA alleles are not necessarily comparable, we considered the rank of each peptide among a set of 100,000 random natural peptides in addition to the prediction score in the peptide-HLA assignment. A peptide was assigned to the HLA isotype (among all HLA isotypes expressed by a given BLCL), for which the peptide showed the highest ranking, while having a predicted binding affinity <5000 nM IC50. In addition, the ranking had to be among the top 5% for HLA-A and -B alleles and the top 10% for HLA-C alleles. We repeated the peptide assignment procedure also based on *either* their predicted binding affinity *or* rank. A peptide would be assigned to an HLA-C molecule only if the peptide did not rank among the top 5% for any of the respective BLCL’s HLA-A or -B isotypes, because the prediction tools for the HLA-C locus do not reach the same prediction performance as for the better-studied HLA-A and -B alleles. HLA assignment was limited to peptides with a length between 8 and 13 amino acids. Peptides that did not meet this length limitation or failed to exceed the prediction score or ranking cut-off were assigned “NA”. For some peptides several length variants were eluted, and these length variants were treated as one instance to avoid any bias during data analyses. Peptides with several length variants were collapsed to the peptide with the best rank and binding affinity score.

We validated our assignment approach by comparing our eluted peptides predicted to be presented by HLA-B*40:01 or -B*4402 with peptides eluted previously by Hillen et al., from BLCL homozygous for these HLA-B alleles [19]. Eighty-six HLA-B*44:02 restricted peptides identified by Hillen et al., [19] were also eluted in our study, of which 83 (96.5%) were indeed assigned to HLA-B*44:02. The remaining 3 HLA-B*44:02 peptides that were also present in our dataset were eluted from a BLCL not expressing HLA-B*44:02 but the closely related HLA-B*40:01 (BLCL 1077). A similar overlap was observed for peptides assigned to HLA-B*40:01, where all of the 27 HLA-B*40:01 restricted peptides identified by Hillen et al., [19] that were also present in our dataset, were indeed assigned to HLA-B*40:01 in our study. Moreover, to investigate to what extend presentation of the same peptide by two or more distinct HLA alleles might influence our results, we reanalysed the data for one of the cell lines (BLCL 1053) also allowing each peptide to bind to as many HLA molecules as it would be predicted to be a binder for. Moreover, we also compared the assignments based on predicted binding affinity alone, and based on rank alone, with the combined approach we used throughout the paper. S2 Fig shows that our peptide assignments are very robust, and that irrespective of the assignment method we always find similar numbers of peptides assigned to HLA class I molecules.

### Gene Ontology analysis

The source proteins associated with the eluted peptides were mapped to the coding genes using UniProt Knowledge database [20]. The annotation of human genes were analysed further with Cytoscape plugin ClueGo [21]. In the cellular location analysis, we used GO categories for cellular location, and reported only the locations that were significantly enriched (p<0.01, after correction for multiple testing using Benjami-Hochberg criteria) compared to the whole human genome. Parameters used were: Kappa = 0.15, GO Term fusion is enabled, GO term restriction is set to minimum level 2, and maximum level 8, minimum number of genes per cluster is 2. The cluster names are given by using the GO category that had maximum number of genes in a cluster. The biological processes clustering was performed similarly. Here all the biological processes that were enriched compared to the whole human genome were reported (p<0.05, after correction for multiple testing using Benjami-Hochberg criteria).

### Protein abundance

Cellular abundance data for 12,021 human proteins, as assembled from public databases, were available from Weiss et al [22]. The abundance is expressed in parts per million (ppm), relative to the molecule counts of all other proteins in the proteome. The measured abundances for different proteins span several orders of magnitude. The data were kindly provided by Christian von MeringUniversity of Zurich.

### Statistical analysis

Plots were made using R (http://www.r-project.org) or GraphPad Prism (Version 5.03), and statistical analysis was performed as indicated. P values <0.05 were considered statistically significant.

## Results

### Characteristics of eluted peptides

**Fig 1:**
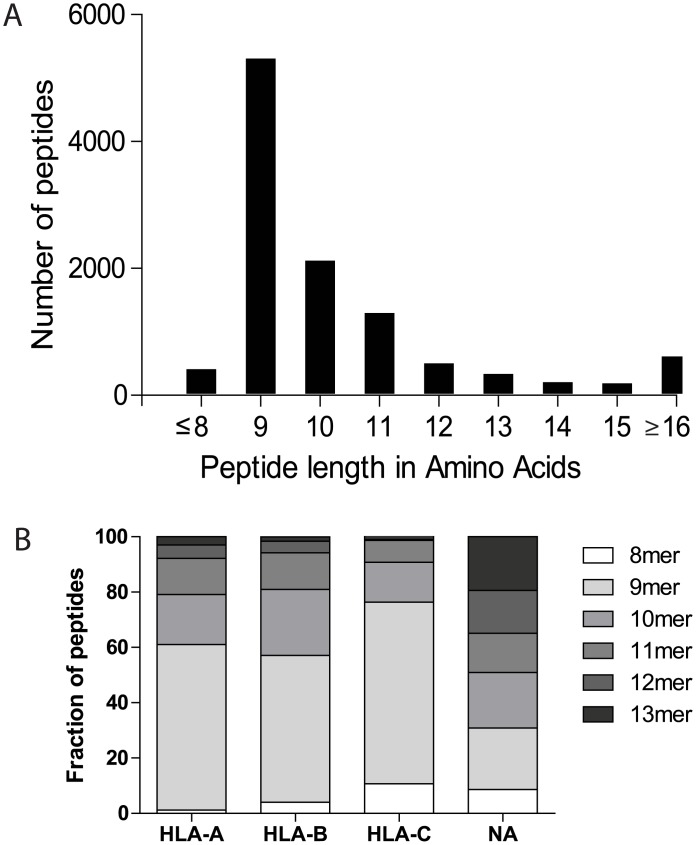
General characteristics of eluted host peptides. Distribution of peptide lengths for all peptides eluted from the four BLCL (A). Peptides ≤8 amino acids are grouped together, as are peptides ≥16 amino acids. Relative distribution of peptide lengths plotted per HLA locus for all peptides eluted from the four BLCL (B). Similar distributions are observed when plotting peptides eluted from individual cell lines. NA: not assigned.

To be able to compare features of naturally processed and presented HLA class I ligands of a broad panel of HLA-A, B and C alleles, the immunopeptidomes from four different BLCL were studied during steady state, or after infection with measles virus (MV). The total number of unique peptides eluted from the four cell lines, in both conditions combined and identified under relatively strict discovery settings, is summarized in Table 1. Altogether, 7897 unique self-peptides, which could be mapped to 4344 human proteins reported in the UniProt database [20], were eluted (see S1 Table. for more details on the eluted peptides). Fig 1A shows the distribution of peptide lengths for all peptides eluted. As expected for HLA class I binding peptides, most of the eluted peptides (53%) are 9 amino acids long and about 90% have a length of 8–13 amino acids, in line with previous results [23].

From the uninfected BLCL altogether, 5767 unique self-peptides were eluted, which were mapped to 3533 human proteins. To better understand the function of the underlying genes and the biological processes involved, we performed a Gene Ontology analysis using the ClueGo plugin in the Cytoscape software [21]. The far majority of identified genes had at least one GO category assigned to them (99.5%). As expected, comparison of the cellular location of genes of which at least one peptide was eluted, with the whole human genome, showed a clear lack of genes associated with the plasma membrane, or extracellular matrix (p<0.001 after correction for multiple testing using Benjami-Hochberg criteria) and an enrichment of intracellular proteins (located in ER, nucleus, cytoplasm and a number of other organelles, p<0.001, after correction for multiple testing using Benjami-Hochberg criteria, results not shown).

### Distribution of eluted peptides over the different HLA molecules

To reveal which of the HLA class I molecules presented the identified peptides, we applied peptide-HLA binding affinity prediction tools (see Methods for details on assignment and validation). For each BLCL, on average, 94% (range 91–97%) of unique peptides, 8 to 13 amino acids in length, could be assigned. Preference in peptide assignment was given to HLA-A and B alleles (see Methods for details). As depicted in Fig 1B, the length distribution is similar for the peptides presented by the different HLA class I loci (p>0.05, Kolmogorov-Smirnov test).

**Fig 2:**
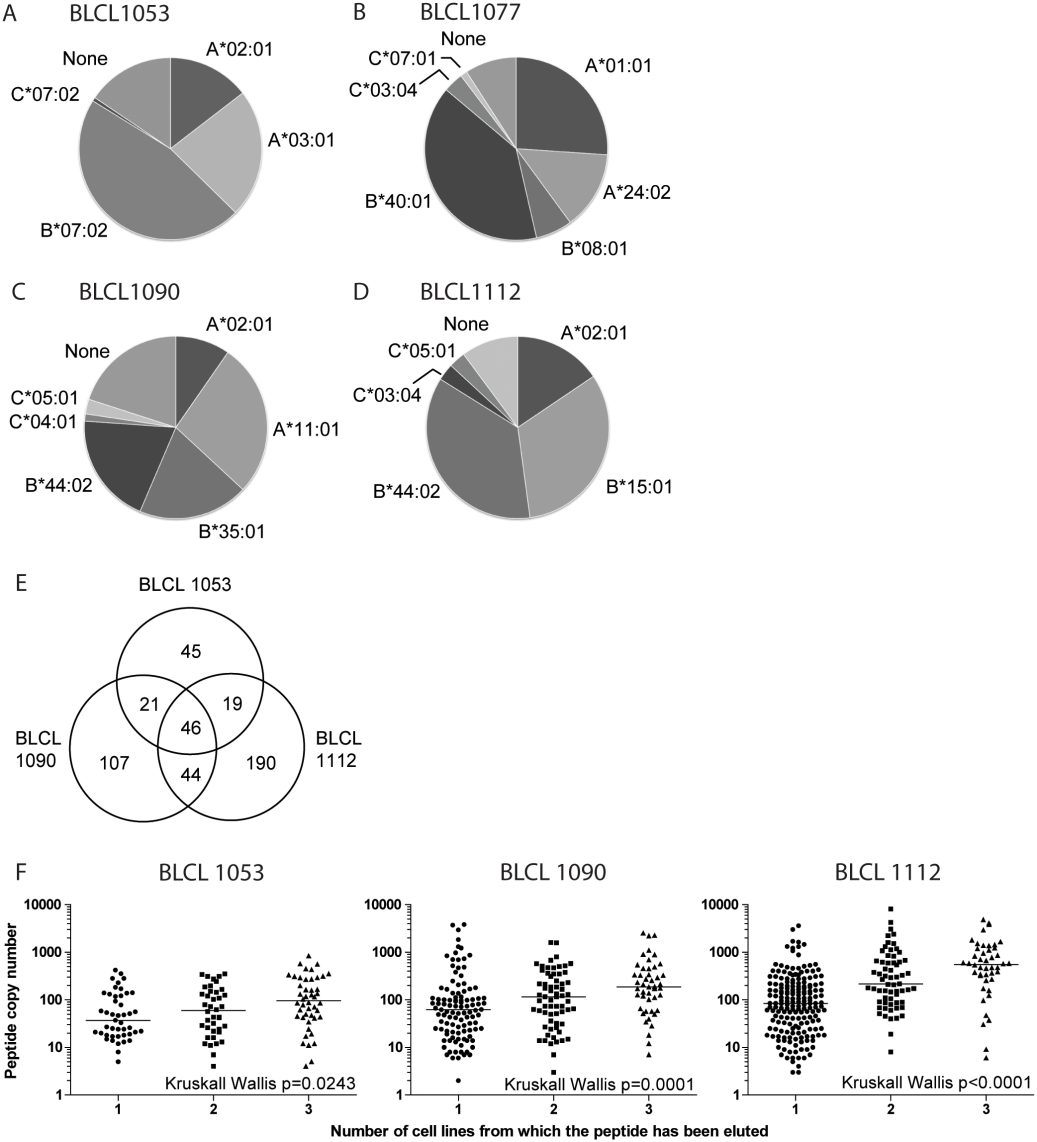
HLA coverage per cell line. The distribution of unique self-peptides eluted from uninfected cells are shown for BLCL 1053 (A), 1077 (B), 1090 (C) and 1112 (D). Each slice of the pie represents the fraction of unique peptides eluted from that specific BLCL presented by the specific HLA molecule. Peptides that could not be reliably assigned to any of the HLA molecules expressed are depicted as **None**. The Venn diagram in (E) reveals the overlap in HLA-A*02:01 binding peptides eluted from the three HLA-A*02:01-expressing BLCL. In (F) the copy number of eluted HLA-A*02:01 peptides is shown for the three HLA-A*02:01-expressing BLCL. The copy number of peptides eluted from only 1 BLCL is significantly lower compared to peptides eluted from ≥2 BLCL (p<0.05, Kruskall Wallis test).

In Fig 2A–2D the distribution of unique peptides per HLA molecule is shown for each of the four uninfected BLCL. Although for each HLA molecule hundreds of unique peptides were eluted (mean 263 unique peptides, range 6–694), there were marked differences in the distribution of the eluted peptides over the different HLA molecules between the four cell lines. For example, whereas the eluted peptides were quite evenly distributed over an HLA-A and B peptidome for BLCL1090 (Fig 2C), the peptides eluted from BLCL1112 were greatly skewed towards being presented by HLA-B molecules (Fig 2D). This might be due to the fact that this cell line is homozygous for HLA-A*02:01 and therefore a smaller fraction of the unique peptides are presented by this HLA allele. This is in contrast, however, to what we observed for BLCL1053, homozygous for HLA-B, where we found that more of the unique peptides were presented by HLA-B*07:02 compared to the two HLA-A alleles together (Fig 2A). The number of eluted peptides predicted to be presented by HLA-C molecules was only a small fraction of the total number of peptides. Although it has been reported that the density of HLA-C molecules on the cell surface is much lower than that of HLA-A and B molecules [24,25], which can explain this discrepancy, we are aware that we most likely underestimate the HLA-C repertoire. Due to lower performance of HLA-C peptide predictions, we however chose to assign a peptide to an HLA-C molecule only if we could not assign that peptide to any of the HLA-A or B molecules expressed by the BLCL.

Three of our BLCL expressed HLA-A*02:01. In total, 472 unique HLA-A*02:01 restricted self-peptides were eluted, of which 72% was found in only one, 18% in two, and 9.7% in all three cell lines (Fig 2E). Interestingly, the HLA-A*02:01 restricted peptides that were found in all three BLCL were present in higher copy numbers compared to peptides eluted from only one or two cell lines (Fig 2F). Similarly, when we compared peptides eluted from the two BLCL expressing HLA-B*44:02, peptides eluted from both cell lines were present in higher copy numbers than peptides eluted only once (p<0.0001, data not shown).

Consequently, we sought to determine factors influencing the relative amount of each peptide. As shown before [26], the predicted MHC binding affinity as well as cellular abundance of the source protein was found to be correlated with the observed copy number of the peptide (Spearmans rho = -0.089, p < 2.2e-16; and Spearmans rho = 0.15, p< 2e-16, respectively, S3 and S4 Figs). The low correlation coefficients suggest that binding affinity and protein abundance are just two of many factors that determine which peptides are more likely to be presented by a cell and therefore, are presented in larger copy numbers per cell and more frequently by different cell lines expressing the same HLA molecule.

### Total number and diversity of self-peptides decreases upon MV infection

**Fig 3:**
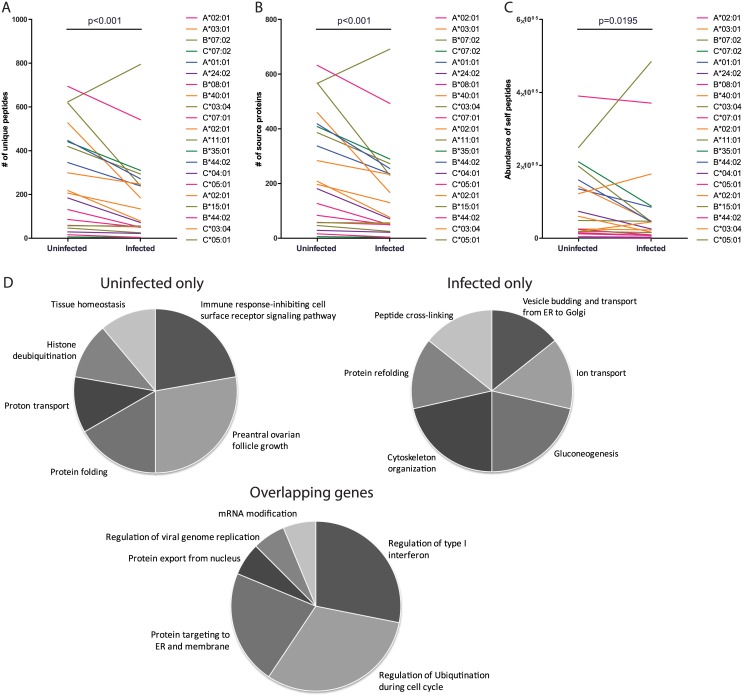
Decrease in host peptides after MV infection. (A) The number of unique peptides is plotted per infection state. Each line represents one HLA molecule expressed on one BLCL. Similarly, in (B) the number of source proteins per HLA molecule per BLCL is depicted, and in (C) the total abundance of pHLA complexes eluted from the BLCL and predicted to be presented by the depicted HLA molecules. Statistical significance of differences between uninfected and infected data sets (A-C) are analysed using the Wilcoxon matched-pairs signed rank test. The pie charts in (D) show GO analysis results of the top 100 most abundantly presented genes grouped for uninfected cells only (n = 45), infected cells only (n = 45), or both (n = 55).

To investigate potential changes in peptide presentation upon viral infection, we additionally eluted peptides from BLCL infected with measles virus. Compared to the total of 5767 unique self-peptides eluted from the four BLCL in steady state, ‘only’ 4112 unique self-peptides were found after MV infection. MV infection thus resulted in a 28.7% decrease in the number of unique peptides derived from self-proteins. Fig 3A shows that the observed decrease was irrespective of the presenting HLA molecule (infected vs uninfected, p = 0.0007). When analysing the data per HLA locus, the decrease was significant for HLA-A and C (p = 0.018 and p = 0.018, respectively) whereas for HLA-B molecules only a trend was observed (p = 0.128), mainly due to an increase in the number of unique peptides eluted from HLA-B*15:01 in BLCL1112. In line with the decrease in the number of unique peptides, also the number of source proteins decreased upon infection, from 3533 to 2604 unique source proteins (depicted per cell line per HLA molecule in Fig 3B), as well as the copy number of these peptides. After normalizing the peptide copy number by the total number of eluted peptides for each of the BLCL this difference was still visible, albeit not statistically significant (data not shown). Altogether, MV infection resulted in a decreased density of HLA presented self-peptides (median number of self pHLA complexes eluted per HLA molecule for uninfected BLCL 48857, range 962–389961; for MV infected BLCL 28540, range 272–488629; p = 0.019; Fig 3C).

### Changes in self source genes upon MV infection

During infection a rather different set of proteins gave rise to eluted self-peptides. The translation or processing of 1387 unique human gene products was apparently lost or altered during the infection, because we were not able to identify peptides derived from these source proteins in infected cells even though they were found in uninfected cells (S2 Table). Alternatively, 635 genes gave rise to peptides uniquely eluted from infected cells (S3 Table), i.e., in uninfected BLCL we did not find any peptides originating from these genes, which suggests an up-regulation of their expression during MV infection. The gene set giving rise to self-peptides presented solely by the infected cells were enriched for genes located intercellularly, e.g., originating from ER, cytoplasm or other organelles and contained a significantly decreased number of proteins located in the extracellular matrix or associated with the membrane, compared to the whole human genome (p<0.01, data not shown). Although the source proteins for peptides eluted from uninfected and infected cells differ, we did not find a clear biological process GO cluster that was significantly enriched or depleted. This might be due to the fact that both uninfected and infected cells in our experiments are EBV-transformed, representing a mutagenized state. Of note, EBV-derived peptides did not occupy much of the available HLA molecules, as only 7 EBV-derived peptides could be found in all four BLCL together (data not shown), in line with [27].

Next, we sorted the source genes according to the total abundance of all eluted peptides derived from these genes, and zoomed into the top 100 genes with the highest amount of peptide supply for peptide presentation. Surprisingly, MV infection has little influence on these most abundantly presented genes, as 55 of the top 100 genes were the same in infected and uninfected cells. The most abundantly represented genes were mainly house-keeping genes playing a role in pathways necessary for maintaining cellular survival, e.g., protein folding and ion transport (Fig 3D). With or without MV infection, the genes involved in the regulation of interferon type I were enriched among the most abundantly represented genes. Of note is also the enrichment of genes involved in cytoskeleton organization following MV infection.

Taken together, our results suggest that upon MV infection of BLCL there is a decrease in the number of self-peptides. This is paralleled by a decrease in the number of source proteins and a shift in the specific genes the presented self-peptides originate from.

### HLA-B molecules present a more diverse repertoire compared to HLA-A molecules

**Fig 4:**
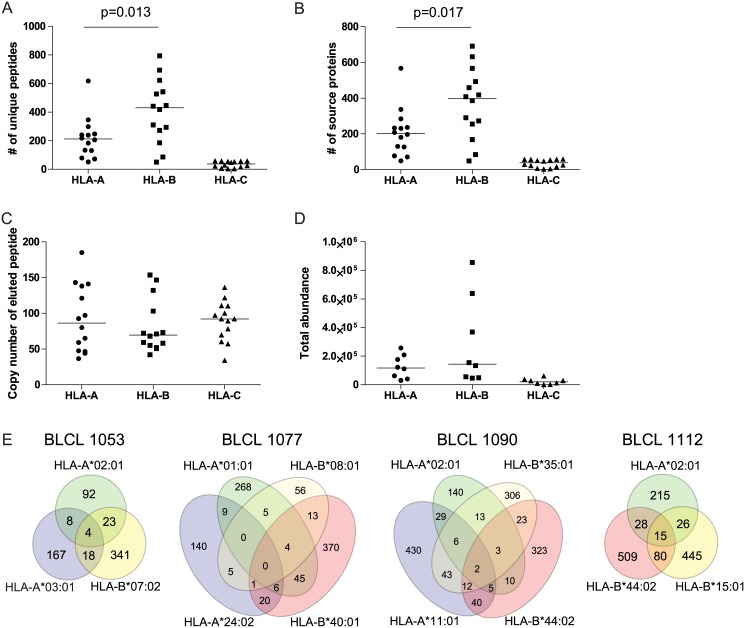
HLA-B molecules present a more diverse self-repertoire than HLA-A molecules. (A) The number of unique self-peptides eluted from HLA-A, HLA-B or HLA-C molecules. Each dot represents one HLA molecule on one BLCL in one infection state. Similarly, (B) represents the number of source proteins the eluted peptides originate from. In (C) the median copy number of eluted peptides is depicted per HLA molecule per BLCL per infection state. (D) shows the total abundance (sum of copy numbers of all peptides for each HLA molecule per BLCL per infection state). Statistical significance of differences between HLA-A and HLA-B data sets (A-D) was analysed using unpaired t testing and only shown if p<0.05. Venn diagrams in (E) show overlap in source protein usage of the eluted self-peptides per HLA molecule for uninfected BLCL. HLA-A molecules are depicted in green and blue, HLA-B molecules in yellow and red. HLA-C molecules are excluded from the statistical (A-D) and Venn diagram analyses (E).

Next we compared the immunopeptidomes of the different HLA-A and HLA-B molecules. Interestingly, we found that in the total dataset from all cell lines, a significantly larger number of unique peptides was assigned to HLA-B molecules than to HLA-A molecules (p = 0.007, Fig 4A). A median number of 430 unique peptides was eluted from HLA-B molecules (range 50–794), compared to a median of 212 unique peptides from HLA-A molecules (range 51–618). S4 Table shows the number of peptides (and percent of total) associated with each HLA molecule in each cell line, with and without MV. In the majority of samples (6 out of 8 experiments), most prominent in BLCL1053 and BLCL1112, an HLA-B allele presented the highest number of unique peptides with the exception of the uninfected BLCL1090, in which HLA-A*11:01 presented the highest number of peptides and the MV-infected BLCL1077, in which HLA-A*01:01 presented the highest number of peptides. The eluted peptides from HLA-B molecules were also derived from a larger number of source proteins compared to HLA-A (p = 0.022, Fig 4B). We observed a tendency that peptides eluted from HLA-A molecules were presented in higher absolute (Fig 4C) as well as normalized (data not shown) copy numbers than peptides eluted from HLA-B molecules (median for peptides eluted from HLA-A molecules 86 copies/cell (range 0–36108, n = 3064 peptides), median for peptides eluted from HLA-B molecules 69 copies/cell (range 0–51639, n = 5683 peptides)). Together, the combination of the increased number of unique peptides found for HLA-B molecules, in somewhat lower copy numbers, results in a similar total abundance (sum of copy numbers of all unique peptides) of pHLA-A and B complexes (Fig 4D).

We next analysed the overlap in source protein usage between HLA molecules expressed on the same BLCL. The Venn diagrams shown in Fig 4E reveal that, in line with our expectations, there is a substantial overlap in source protein usage by the different HLA molecules. On average 24% (range 11,7–34,9%) of all source proteins were targeted by at least two different HLA molecules expressed on the same cell line. For BLCL1090, there were even two source proteins (F2Z2U4: Transformation/transcription domain-associated protein, and SYNE2: Nesprin-2) that were targeted by all four HLA molecules, and 26 source proteins targeted by three out of four HLA molecules.

Taken together, our results based on BLCL-derived HLA class I eluates indicate that the studied HLA-B molecules in general presented a more diverse repertoire of self-derived peptides compared to the HLA-A molecules, which could play a role in their immunodominance.

## Discussion

It is suggested that HLA-B molecules have a superior impact on the outcome of several infectious diseases, inflammatory conditions, autoimmune diseases and malignancies compared to HLA-A molecules, for reasons that remain not well understood. To our knowledge this is the first large scale mass spectrometry-based study to investigate broadly, using 5 HLA-A and 6 HLA-B molecules, whether there are typical features of the naturally presented immunopeptidome that distinguish HLA-B molecules from their HLA-A counterparts, which might be related to the association between HLA-B molecules and disease outcome. Indeed we found evidence for a certain “HLA-B-*ness*” versus “HLA-A-*ness*” of HLA class I self-peptide repertoires, possibly contributing to the immunodominance of the HLA-B molecules. While no significant locus specific differences were found in peptide length distributions, total peptidome abundances or predicted affinities, we saw an increased overall number of unique self-peptides and source proteins in the immunopeptidome associated with the studied HLA-B alleles compared to the HLA-A alleles. One could argue that by presenting a broader peptide repertoire, HLA-B molecules simply have a bigger chance of presenting immunodominant peptides. This might provide an explanation why HLA-B molecules have been associated with dominant immune responses more often.

In our study, HLA-A02:01 was present in 3 out of 4 investigated cell lines, however, we feel that this overrepresentation did not influence our results as HLA-A02:01 did not behave differently in our analyses. Moreover, the observed trend that HLA-B molecules present a more diverse repertoire than HLA-A molecules is in line with a recent other comprehensive study investigating the self-peptide repertoire of a human BLCL, expressing HLA-A1, HLA-A3, HLA-B7 and HLA-B27 [28]. In this study, 9% and 17% of all peptides eluted were assigned to HLA-A1 and -A3, respectively, whereas 35% and 29% of the eluted peptides were assigned to HLA-B7 and -B27, respectively. Further research is needed to gain insight into a potential role for certain individual HLA-A and B- alleles and the cellular origin for the observed trend in diversity of HLA-A- versus -B- presented peptide repertoires. To see how specific our results are for the experimental setup and the computational approach we have used, we reanalysed the data of other groups that reported self-peptide repertoires on a less comprehensive scale from primary tissue of various HLA-A- and -B- heterozygous backgrounds.[29,30] Unfortunately, we did not observe the same HLA-based peptide presentation skewing when analysing HLA-A versus HLA-B presentation in these data sets (data not shown). Differences in the number of eluted peptides, HLA binding prediction methods and HLA assignments, presence of certain HLA molecules and cell and tissue backgrounds may all have potentially influenced the obtained results. Notably, in our own previous study, we analysed the diversity and binding affinity of the peptide repertoires of HLA-A and B molecules by making use of curated pathogen-derived epitope data retrieved from the Immune Epitope Database and Analysis Resource (IEDB), including *in silico* predicted epitopes, and found that HLA-B molecules presented a peptide repertoire that was less diverse and of a lower binding affinity compared to HLA-A molecules [31]. The difference between our current work and our previous results was not due to a difference in the HLA molecules that were included, as repeating the previous analysis only for the HLA molecules in the present study resulted in similar findings (results not shown). This suggests that even though the repertoire of potential peptides present in the ER fitting the binding motif for HLA-A molecules seems larger compared to HLA-B molecules, the actual repertoire of naturally processed and presented peptides is possibly largest for HLA-B molecules. This might be due to differences in the stability of the pMHC complexes, which has been shown to be important for immunogenicity [32–34]. Hence it would be of interest to compare the stability of natural pHLA-A complexes and pHLA-B complexes in future studies. Differences in stability between the two loci can result in a more diverse peptide repertoire presented by HLA-B molecules if fewer pHLA-A complexes are stable enough to be presented at the cell surface.

We used MV as a model pathogen in this study. After infection, the overall number and diversity of self-peptides eluted decreased, largely irrespective of the presenting HLA allele. This is not unexpected, as normal cell metabolism is affected and viral peptides may now also occupy part of the available HLA molecules [35]. When taking into account the copy numbers of MV derived peptides (Schellens *et al*., unpublished data), we still observed a decrease in total abundance of pHLA complexes in two out of four BLCL upon MV infection. MV did however not downregulate the overall expression of MHC class I expression at the cell surface (S1 Fig), a known immune evasion strategy for many other viruses (reviewed by e.g. [36,37]). Cellular stress conditions imposed by a viral infection may lead to a reduced supply of peptides owing to an inhibition of overall protein synthesis, or to an altered quality control of the MHC class I peptide cargo of the cell [38,39]. As a consequence, the generated peptide repertoire under these MV induced stress conditions may contain less affine binders and/or an increased proportion of unstable MHC peptide complexes [40], which may not survive our peptide work-up conditions. Whether MV infection interferes with components of the peptide loading machinery of cells is to our knowledge unknown and remains to be investigated. In agreement with this hypothesis, we compared the predicted binding affinities of the self-peptides eluted from non-infected and infected cells, and found that the peptides eluted from non-infected cells had a slightly (though significant) higher predicted affinity than the peptides eluted from the infected cells (results not shown).

Our study additionally gave unique insights into the outcome of the processing machinery under various conditions. Our GO analysis revealed that genes of which at least one peptide is eluted, are mostly encoding for intracellular proteins, and that the most abundantly presented genes are mainly house-keeping genes playing a role in pathways necessary for cell survival. Constitutive expression of these vital genes may also underlie the large overlap in the top 100 most abundantly presented genes observed for infected and uninfected cells. These data are in line with previous studies analysing sources of HLA class I ligands [41–43].

As previously observed, there is a marked difference in the copy number of eluted class I peptides [11,27,44–46]. In our dataset, peptide abundance could be partially explained by the cellular abundance of that peptide’s source protein and the predicted binding affinity of that peptide in the MHC complex (with better binding resulting in more copies of the peptide). But, as these correlations were rather weak, these factors could explain only part of the observed variance in copy numbers. Interestingly, we found that peptides eluted from 2 or 3 different BLCL were present in higher copy numbers compared to peptides eluted from only one cell line (Fig 2F and data not shown). Hassan et al. also reported the relation between the abundance of an epitope and the chance of being identified in a MHC class I eluate [27]. The biological relevance of the observed differences in copy numbers is however unknown, as it has been shown that CD8+ T cells can already recognize and respond to as few as one pHLA complex on the surface of a target cell [47]. On the other hand, very high epitope density may drive a vigorous primary T cell response, but could also result in clonal exhaustion [48]. As it is clear from the study by Hassan et al [27] and ours, the most abundant self-epitopes are easiest to be identified with certainty by current nanoLC-mass spectrometry systems. Since undersampling is unavoidable in this system, epitopes present at lower copy numbers may be outcompeted more easily. Continuous technical refinement of separation systems and MS/MS will be needed to get a closer look at the low-end of the dynamic range of HLA class I peptidomes.

Studies investigating the repertoire of naturally processed and presented epitopes have often been limited to single HLA class I molecules [49–51], sometimes making use of recombinant soluble HLA class I molecules normally not present on the specific cell line [52,53]. Although there are several benefits of such an approach, these systems do not reflect the natural system where each cell expresses 3 to 6 different HLA class I molecules, which might influence the presented repertoire. However, HLA-transfected cell lines could be valuable tools to follow up on observations reported in the present paper, e.g. by confirming features of peptide diversity of a particular HLA-B allele in the presence or absence of other HLA-A- or B alleles.

Here we show that identification of naturally processed and presented peptides for a broad panel of HLA molecules provides unique insights into the complex system of antigen presentation. The studied peptidomes showed that i) there are marked differences in the number of unique peptides presented by different HLA molecules, ii) peptides eluted from more than one cell line are present in higher copy numbers compared to peptides that are found only in a single cell line, iii) the diversity and abundance of self-peptides decreases upon MV infection, iv) presenting a more diverse peptide repertoire is a feature of certain HLA-B alleles compared to HLA-A alleles. Whether the latter observed tendency is broadly applicable to the HLA-B locus and contributes to the differential impact of HLA-B alleles on the outcome of certain disease needs further investigation.

## Supporting Information

S1 Fig Cell surface expression of HLA class I on uninfected and MV infected BLCL. 2x10^6^ uninfected and 48 hr MV infected BLCL were analysed by FACS staining as indicated for MV-H expression and for expression of total HLA class I, HLA-A2 and HLA-B,C. Shown are overlays of cell surface expression of indicated markers on uninfected (white plots) and MV infected (grey plots) BLCL1053 (HLA class I alleles: A*02:01, A*03:01, B*07:02; upper panel) and BLCL1112 (HLA class I alleles: A*02:01, B*15:01, B*44:02; lower panel). Results are representative for other BLCL tested (n = 3) (data not shown).
(TIF)

S2 Fig Comparison of different assignment strategies. The number of unique self peptides predicted to be eluted from HLA-A, HLA-B or HLA-C molecules was plotted for BLCL 1053. Each dot represents a different assignment strategy, based on either i) predicted affinity and rank, as described in the materials and methods section and used throughout the paper (red circles), or ii) predicted binding affinity, the peptide was assigned to the HLA molecule with the best IC50 (black triangles), or iii) rank, the peptide was assigned to the HLA molecule with the highest rank (black squares), or iv) proportional approach, the peptide was proportionally assigned to the HLA molecules it was predicted to be a binder for (open squares).
(PDF)

S3 Fig Correlation between predicted MHC binding affinity and observed copy number. For each of the eluted peptides, the observed copy number (depicted as percentage abundance on the cell surface, x-axis) was plotted against the predicted binding affinity (y-axis). To this end, the rank of each peptide among a set of 100,000 random natural peptides (depicted as a percentage) was used as a measure of binding affinity. A significant correlation was observed, spearmans rho = -0.089, p<2.2e-16.
(PDF)

S4 Fig Correlation between cellular abundance of the source protein and observed copy number. For each of the four cell lines cellular abundance of the source protein (in Log10, x-axis) was plotted against the observed copy number of the eluted peptide (in Log10, y-axis). On the left hand uninfected cell lines are depicted, on the right hand infected cell lines. All correlations were tested using Spearmans correlation test. 1053,0 (uninfected): spearmans rho = 0.1145779, p = 0.00866; 1053,1 (infected): spearmans rho = 0.07058086, p = 0.1839; 1077,0 (uninfected): spearmans rho = 0.1420078, p = 9.526e-05; 1077,1 (infected): spearmans rho = 0.1649588, p = 0.0008612; 1090,0 (uninfected): spearmans rho = 0.2136391, p = 1.319e-13; 1090,1 (infected): spearmans rho = 0.2278638, p = 1.163e-09; 1112,0 (uninfected): spearmans rho = 0.06460327, p = 0.03279; 1112,1 (infected): spearmans rho = 0.1745593, p-val = 2.245e-08.
(PDF)

S1 Table List of peptide sequences. For each eluted peptide, the following information is included in the table: name of the cell line, infection status, epitope sequence, epitope length, estimated copy number, source protein and gene, most likely HLA allele presenting the peptide based on prediction score or rank, normalized copy number.
(XLSX)

S2 Table Human genes down-regulated during MV infection. Peptides derived of 1387 unique human genes were found solely in uninfected cells. For each of the genes, the HGNC Approved name and ID are listed, as well as their chromosomal location (HGNC Database, HUGO Gene Nomenclature Committee (HGNC), EMBL Outstation—Hinxton, European Bioinformatics Institute, Wellcome Trust Genome Campus, Hinxton, Cambridgeshire, CB10 1SD, UK www.genenames.org. Analyzed in June 2013).
(XLSX)

S3 Table Human genes up-regulated during MV infection. Peptides derived of 635 unique human genes were found solely in infected cells. For each of the genes, the HGNC Approved name and ID are listed, as well as their chromosomal location (HGNC Database, HUGO Gene Nomenclature Committee (HGNC), EMBL Outstation—Hinxton, European Bioinformatics Institute, Wellcome Trust Genome Campus, Hinxton, Cambridgeshire, CB10 1SD, UK www.genenames.org. Analyzed in June 2013).
(XLSX)

S4 Table Number of peptides (and percent of total) associated with each HLA molecule in each cell line, with and without MV. For each cell line the absolute number of eluted peptides assigned to each of the HLA class I molecules is shown in infected and uninfected cells. NA: not assigned.
(XLSX)
