# Supplementary figures and images for: Comprehensive Analysis of the Naturally Processed Peptide Repertoire: Differences between HLA-A and B in the Immunopeptidome
Source: PLoS One. 2015 Sep 16;10(9):e0136417. doi: 10.1371/journal.pone.0136417 (PMC4574158; doi:10.1371/journal.pone.0136417)

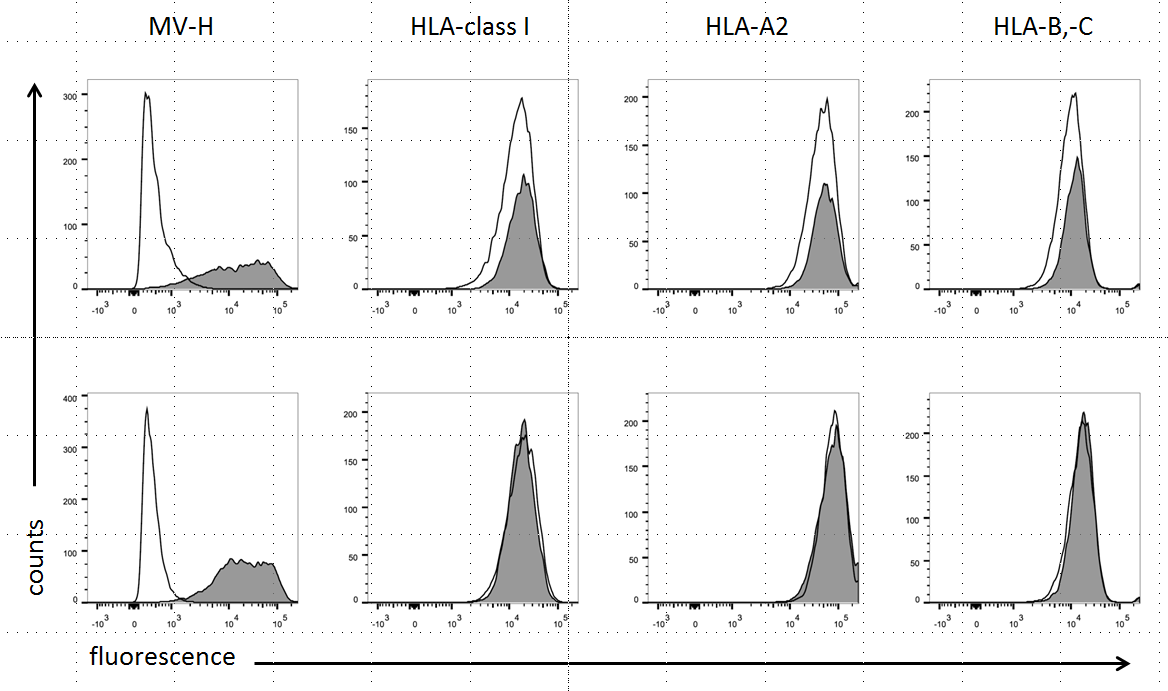

Supplement: S1 Fig — 2x10^6^ uninfected and 48 hr MV infected BLCL were analysed by FACS staining as indicated for MV-H expression and for expression of total HLA class I, HLA-A2 and HLA-B,C. Shown are overlays of cell surface expression of indicated markers on uninfected (white plots) and MV infected (grey plots) BLCL1053 (HLA class I alleles: A*02:01, A*03:01, B*07:02; upper panel) and BLCL1112 (HLA class I alleles: A*02:01, B*15:01, B*44:02; lower panel). Results are representative for other BLCL tested (n = 3) (data not shown). (TIF) [file pone.0136417.s001.tif]

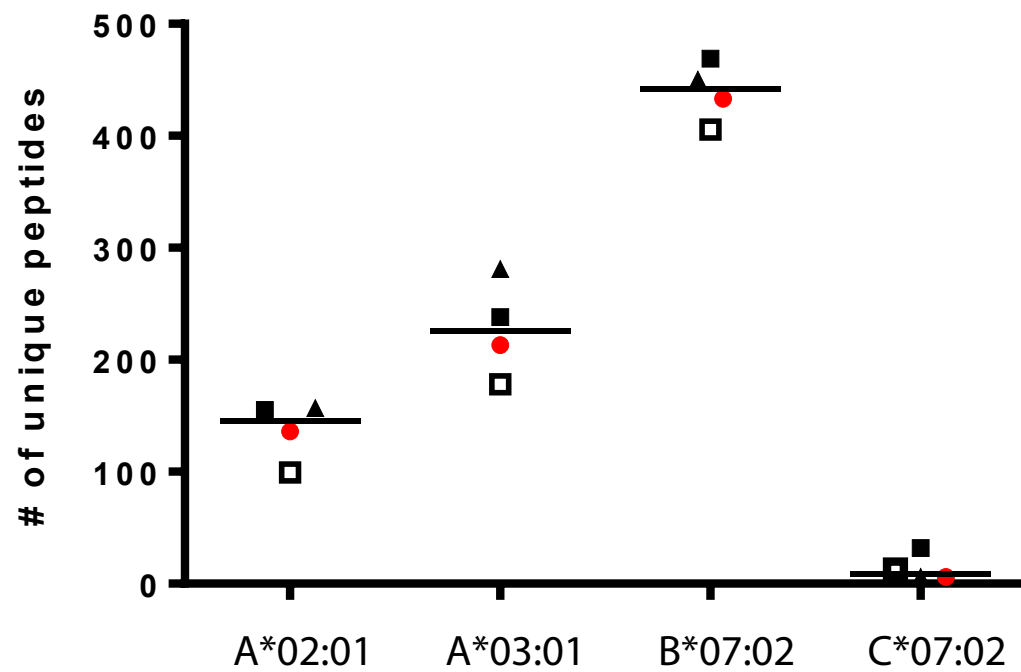

Supplement: S2 Fig — The number of unique self peptides predicted to be eluted from HLA-A, HLA-B or HLA-C molecules was plotted for BLCL 1053. Each dot represents a different assignment strategy, based on either i) predicted affinity and rank, as described in the materials and methods section and used throughout the paper (red circles), or ii) predicted binding affinity, the peptide was assigned to the HLA molecule with the best IC50 (black triangles), or iii) rank, the peptide was assigned to the HLA molecule with the highest rank (black squares), or iv) proportional approach, the peptide was proportionally assigned to the HLA molecules it was predicted to be a binder for (open squares). (PDF) [file pone.0136417.s002.pdf]

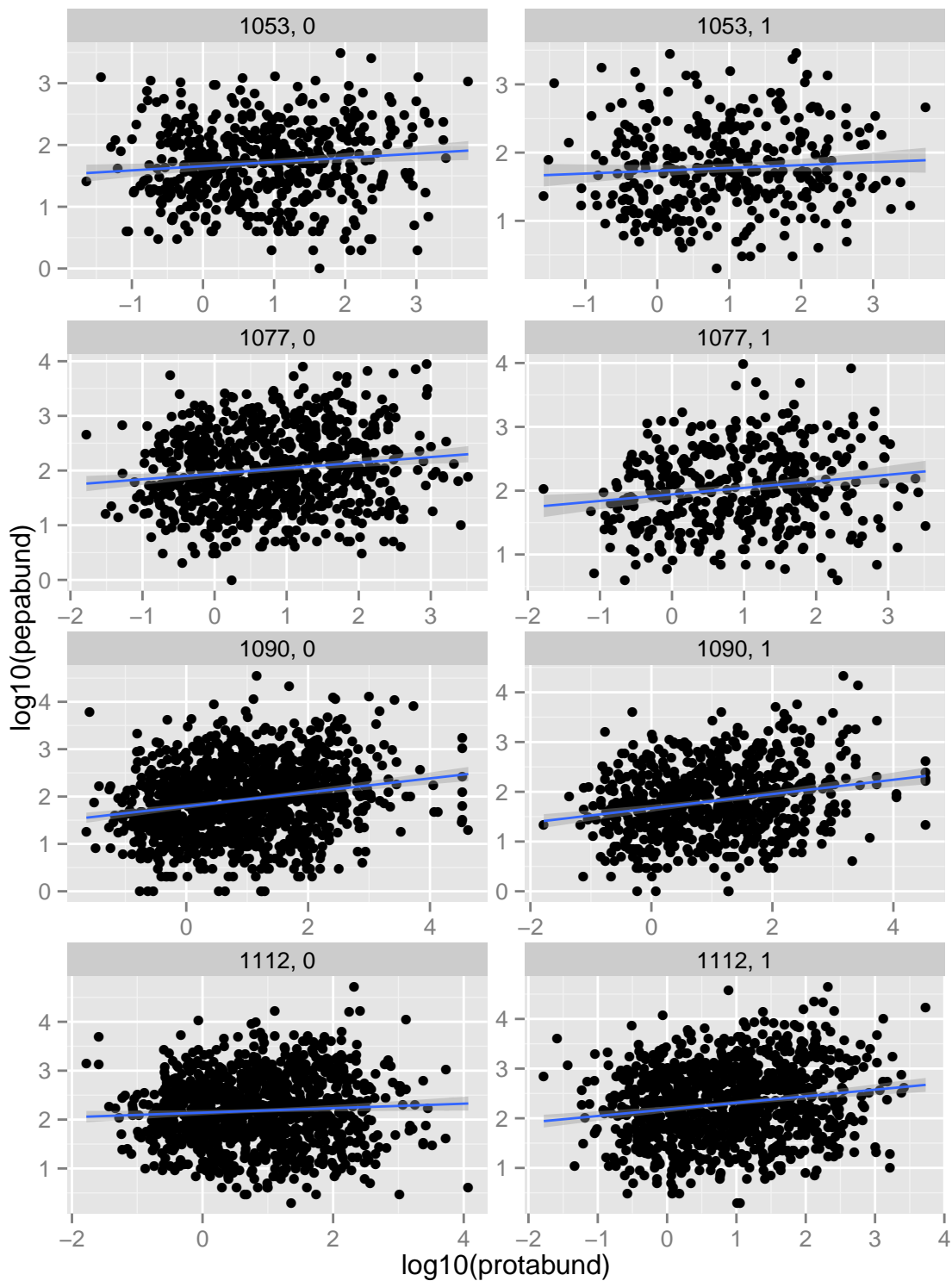

Supplement: S3 Fig — For each of the eluted peptides, the observed copy number (depicted as percentage abundance on the cell surface, x-axis) was plotted against the predicted binding affinity (y-axis). To this end, the rank of each peptide among a set of 100,000 random natural peptides (depicted as a percentage) was used as a measure of binding affinity. A significant correlation was observed, spearmans rho = -0.089, p<2.2e-16. (PDF) [file pone.0136417.s003.pdf]

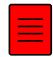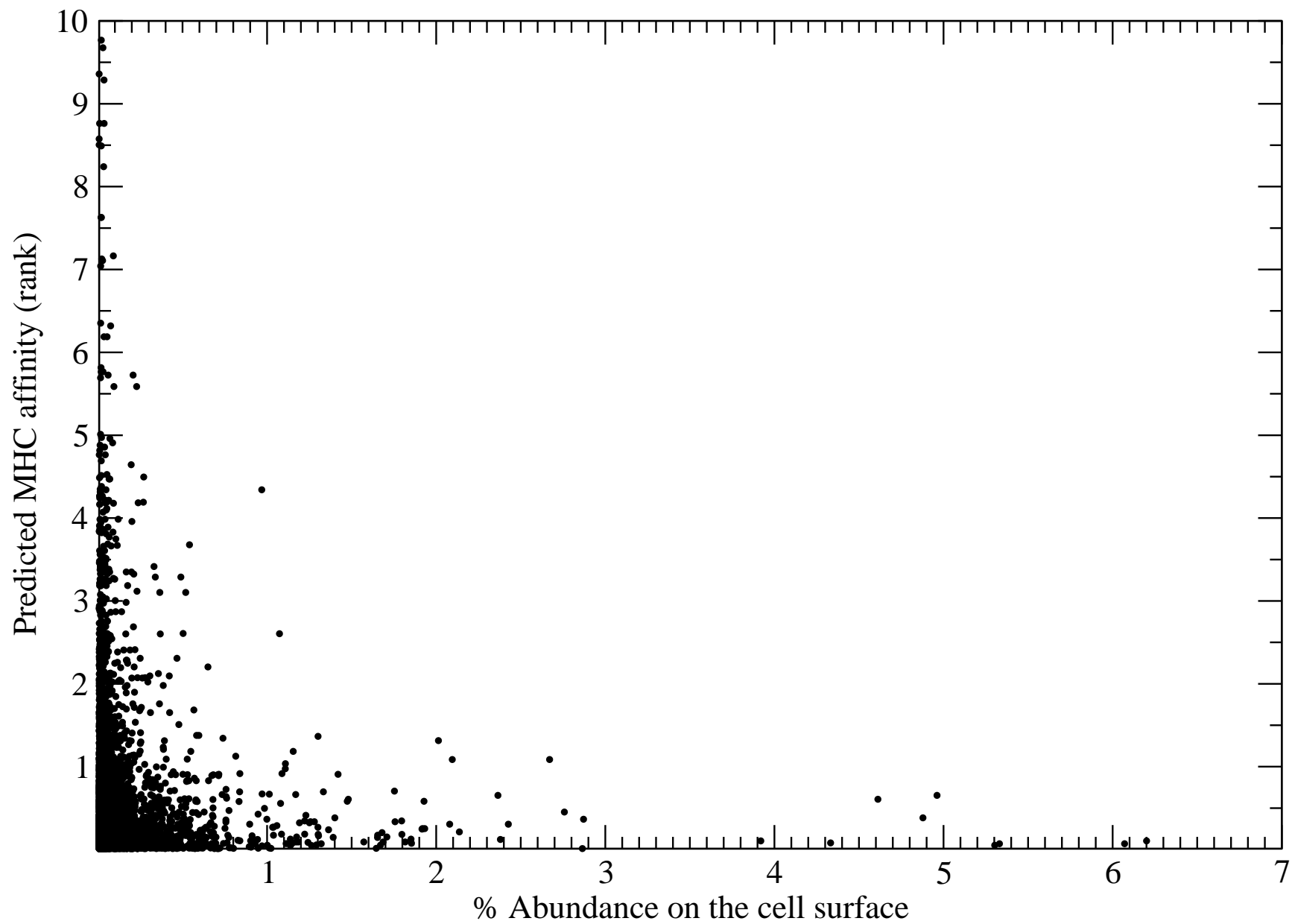

Supplement: S4 Fig — For each of the four cell lines cellular abundance of the source protein (in Log10, x-axis) was plotted against the observed copy number of the eluted peptide (in Log10, y-axis). On the left hand uninfected cell lines are depicted, on the right hand infected cell lines. All correlations were tested using Spearmans correlation test. 1053,0 (uninfected): spearmans rho = 0.1145779, p = 0.00866; 1053,1 (infected): spearmans rho = 0.07058086, p = 0.1839; 1077,0 (uninfected): spearmans rho = 0.1420078, p = 9.526e-05; 1077,1 (infected): spearmans rho = 0.1649588, p = 0.0008612; 1090,0 (uninfected): spearmans rho = 0.2136391, p = 1.319e-13; 1090,1 (infected): spearmans rho = 0.2278638, p = 1.163e-09; 1112,0 (uninfected): spearmans rho = 0.06460327, p = 0.03279; 1112,1 (infected): spearmans rho = 0.1745593, p-val = 2.245e-08. (PDF) [file pone.0136417.s004.pdf]
